# Molecular epidemiology of the expression of urokinase plasminogen activator receptor-associated protein (uPARAP) in mesenchymal malignancies

**DOI:** 10.1016/j.tranon.2026.102708

**Published:** 2026-02-23

**Authors:** Chao-Chi Wang, Pernille Barkholt, Agnieszka Wozniak, Ulla Vanleeuw, Che-Jui Lee, Luna De Sutter, Lore De Cock, Kimberly Verbeeck, Lars Henning Engelholm, Carmel Lynch, Dominik Mumberg, Patrick Schöffski

**Affiliations:** a Laboratory of Experimental Oncology, Department of Oncology, KU Leuven, Leuven Cancer Institute, Leuven, Belgium; b Adcendo ApS, Copenhagen, Denmark; c Department of General Medical Oncology, University Hospitals Leuven, Leuven Cancer Institute, Leuven, Belgium

**Keywords:** Soft tissue sarcoma, Bone sarcoma, uPARAP, Endo180, Therapeutic target, Tissue microarray, Immunohistochemistry

## Abstract

**Introduction:**

Sarcomas are highly heterogenous and rare malignant tumors derived from mesenchymal cells. The current standard treatments for advanced disease have low response rates and are typically associated with considerable toxicity. The aim of this study was to investigate the expression of a potential novel therapeutic target and its clinical correlation in sarcomas, namely urokinase plasminogen activator receptor-associated protein (uPARAP/Endo180/CD280).

**Materials and Methods:**

We evaluated uPARAP expression in various sarcoma subtypes and in normal tissues using 12 tissue microarrays. Expression was assessed on immunohistochemically stained slides and scored according to staining intensity and the percentage of positive tumor cells. Clinical correlations between uPARAP expression and selected parameters (gender and sample origin) were analyzed using generalized estimating equations, and survival outcomes were evaluated with Kaplan-Meier and log-rank tests.

**Results:**

Our results demonstrated limited uPARAP expression in normal tissues, while high expression was observed in the majority of analyzed sarcoma subtypes, particularly high (>80% of highly positive cases) in fibrosarcoma, synovial sarcoma, undifferentiated pleomorphic sarcoma, malignant peripheral nerve sheath tumor, and various bone sarcomas. Based on the correlation analyses between uPARAP expression and selected clinical parameters or survival outcomes, most subtypes did not show a statistically significant association.

**Conclusion:**

In conclusion, this study highlights the potential of uPARAP as an innovative target for targeted treatments, such as novel antibody-drug conjugates in sarcoma.

## Highlights

• uPARAP expression was assessed in sarcoma samples and normal tissues using tissue microarrays.
• High uPARAP expression was observed in the majority of sarcoma subtypes analyzed.
• uPARAP showed limited expression in normal tissues.
• Most sarcoma subtypes did not show statistically significant associations between expression and selected clinical parameters or survival outcomes.
• uPARAP is a highly promising target for therapies such as antibody-drug conjugates.

**List of abbreviations**
ADC: Antibody-drug conjugate
AS: Angiosarcoma
BS: Bone sarcoma
DDLPS: Dedifferentiated liposarcoma
H&E: Hematoxylin and eosin
IHC: Immunohistochemistry
LMS: Leiomyosarcoma
MFS: Myxofibrosarcoma
MPNST: Malignant peripheral nerve sheath tumor
MRCLS: Myxoid round cell liposarcoma
OS: Overall survival
PFS: Progression-free survival
PLS: Pleomorphic liposarcoma
RMS: Rhabdomyosarcoma
STS: Soft tissue sarcoma
SynSa: Synovial sarcoma
TMA: Tissue microarray
uPARAP: Urokinase plasminogen activator receptor-associated protein
UPS: Undifferentiated pleomorphic sarcoma
UZ Leuven: University Hospitals Leuven
WDLPS: Well-differentiated liposarcoma

## Introduction

Sarcomas are a highly heterogenous group of very rare malignant tumors originating from mesenchymal precursor cells. According to the current World Health Organization classification there are >100 distinct subtypes of sarcomas [1,2]. Sarcomas can be generally divided into two main categories, soft tissue sarcomas (STS) and bone sarcomas (BS). The estimated overall incidence of adult STS (excluding gastrointestinal stromal tumors) is about 4–5 cases per 100,000/year in Europe, while for BS it is about 0.8–0.9 cases per 100,000/year across all age groups [[3], [4], [5], [6]]. Most primary sarcomas are treated surgically, some with multimodal therapies combining surgery, radiotherapy and/or systemic chemotherapy. Systemic therapy (chemotherapy and some targeted agents) is mainly used in STS patients with advanced, inoperable and/or metastatic disease, in most cases with palliative treatment intent. Doxorubicin is a common standard of care for many sarcomas, despite low response rates, considerable toxicity and poor survival outcomes [1,7]. There is a high unmet medical need to develop and evaluate new experimental compounds in patients with advanced STS and to explore new, promising biological targets and novel agents, with the long-term goal to improve disease control, survival outcomes and the quality of life of sarcoma patients.

The urokinase plasminogen activator receptor-associated protein (uPARAP, also known as Endo180 or CD280) is the product of the *MRC2* gene. uPARAP is a collagen receptor that plays an important role in collagen internalization and degradation in cells [8]. As collagen is one of the major components of the extracellular matrix of various tissues [9], and since degradation of the extracellular matrix is a crucial step in tumor invasion and metastasis, uPARAP also plays a critical role in various types of cancer and may contribute to cancer progression [10]. In healthy tissue, uPARAP has a limited distribution and is mainly expressed in certain subsets of cells of mesenchymal origin, such as fibroblasts, which play a role in bone development [8,11]. However, uPARAP is also known to be highly expressed in cancer-associated fibroblasts, where the receptor can be present on the cell membrane, in cytoplasmic endosomes, and/or in the perinuclear region [12]. Additionally, expression has been observed in certain subsets of tumor cells, such as in glioblastoma, some breast cancers, mesothelioma, and sarcoma [8,[13], [14], [15], [16], [17]]. Previous studies reported that uPARAP can be found in many STS subtypes, such as in dedifferentiated liposarcoma (DDLPS), fibrosarcoma, synovial sarcoma (SynSa), rhabdomyosarcoma (RMS), leiomyosarcoma (LMS), undifferentiated pleomorphic sarcoma (UPS) and well-differentiated liposarcoma (WDLPS). High levels of uPARAP expression were also found in osteosarcoma [14,17,18]. Furthermore, uPARAP exhibits relatively rapid internalization, which makes it an ideal carrier for targeted therapies[19]. As a result, uPARAP is considered a potential therapeutic target in some malignancies [10,20,21].

In the context of identifying novel targets and developing innovative therapies for sarcoma, we were interested in exploring in more detail the molecular epidemiology of uPARAP expression across a broad range of STS and BS, as well as its correlation with selected clinical parameters.

## Materials and methods

### Normal tissue and sarcoma tissue microarrays

For this study, 8 ready-to-use STS tissue microarrays (TMAs) from KU Leuven and the University Hospitals Leuven (UZ Leuven) and 4 commercially available TMAs from TissueArray and Super BioChip were used. For this retrospective study, the use of archival tumor samples from STS patients for TMA construction at KU Leuven/UZ Leuven was approved by the Ethics Committee Research UZ/KU Leuven (reference number: S68068), and additional approval was granted for the secondary use of tumor samples for the purpose of this study (S66008).

STS TMAs from KU Leuven/UZ Leuven consisted of tumor samples collected between March 2017 and September 2021 from a total of 320 individual patients, with more common subtypes of STS, namely LMS, DDLPS, myxoid round cell liposarcoma (MRCLS), pleomorphic liposarcoma (PLS), WDLPS, angiosarcoma (AS), malignant peripheral nerve sheath tumor (MPNST), myxofibrosarcoma (MFS), RMS, SynSa, and UPS. The original donor tumor blocks were sectioned and stained with hematoxylin and eosin (H&E). Areas of interest were selected microscopically and reviewed by a reference sarcoma pathologist (Dr. Raf Sciot, UZ Leuven). Cores were then obtained from different tumor regions, and each TMA consisted of two to three tumor tissue cores (diameter of 1.0 mm) per sample. From 66 patients more than one sample was collected, either from different sites of disease and/or from different treatment time points (e.g., primary diagnosis, local relapse, or metastasis). The construction procedure of the TMAs was previously described by Lee et al. [22].

Furthermore, three commercially available TMAs from TissueArray were also used, one fibrosarcoma TMA (SO961), one osteosarcoma TMA (OS208a), and one TMA containing multiple normal tissue samples representing normal human organs (FDA999w1). In addition, one commercial TMA containing various STS subtypes and healthy tissues from Super BioChip (NBP2–30,332) was used. Detailed information about these TMAs can be found on the corresponding company websites (TissueArray: https://www.tissuearray.com/tissue-arrays; Super BioChip: https://www.tissue-array.com/main.html).

### Immunohistochemistry (IHC) staining

To evaluate the uPARAP expression in different STS subtypes, IHC staining was performed on 4–5 μm sections cut from constructed TMA blocks. IHC was performed on the Ventana Discovery Ultra automated platform using a standard protocol: heat-induced epitope retrieval was performed in Discovery Cell Conditioning solution 2 (pH 5.20–6.20) for 16 min at 97 °C, followed by incubation for one hour at room temperature with primary antibody (mouse anti-MRC2 [OTI9G4], OriGene, cat. no. TA811858) diluted 1:40,000. Visualization was performed with OmniMap anti-mouse HRP (Roche, cat. no. 760–4310) followed by ChromoMap DAB (Roche, cat. no. 760–159). Finally, sections were counterstained with Hematoxylin II (Roche, cat. no. 790–2208).

### Histological assessment

uPARAP expression was assessed on IHC stained sections by two independent pathologists (Dr. Anand Chainsukh Loya, Sarcoma specialist at Department of Pathology, Copenhagen University Hospitals, and Dr. Paul Snyder, a senior pathologist at Experimental Pathology Laboratories, EPL Inc., Virginia and North Carolina, United States). Staining was scored according to the percentage of uPARAP positive tumor cells and the expression intensity (0, negative; 1, weak; 2, moderate; 3, strong). For duplicate and triplicate samples, the average percentage and intensity were calculated. TMA cores with missing tissue or with more than half of the core lost were excluded from the analysis. Based on the intensity and the percentage of positive tumor cells, tumors were grouped into three uPARAP expressing subgroups, uPARAP negative tumors with <5 % positive tumor cells, low expressing tumors with intensity score 1 and/or <50 % positive tumor cells, and highly expressing tumors with intensity score 2 or 3 and >50 % positive tumor cells.

### Statistical analysis

The sarcoma samples from KU Leuven/UZ Leuven were analyzed for clinical correlations between uPARAP expression and selected clinical parameters, including gender of the donor and sample origin (primary tumor, local relapse, or metastasis).

Samples collected from different sites or at different time points from the same patient were also taken into consideration. Therefore, due to multiple sampling and repeated measurements, generalized estimating equations were used. Individual patient identifiers were considered as subject variables (repeated measures), uPARAP expression level (high, low, or negative) as the dependent variable (response), and gender and sample origin as independent variables (predictors). The Fisher’s scoring method was used for parameter estimation [23], and an ordinal logistic model was applied for the analysis.

Overall survival (OS) of the tissue donors was calculated from the date of diagnosis of sarcoma to the date of death. Patients who were still alive or lost to follow-up were censored on the date of last follow-up. Progression-free survival (PFS) was calculated from the date of diagnosis to the date of first local recurrence and/or metastasis. Patients with no disease progression or relapse were censored on the date of death or last follow-up. Patients with synchronous metastases at diagnosis were excluded from the analysis, to focus solely on patients with localized disease at baseline. Data were analyzed using the Kaplan-Meier method and compared with the log-rank (Mantel-Cox) test.

Statistical analyses and graph plotting were performed using GraphPad Prism (GraphPad Software Corporation, version 10.4.2 [633]) and Statistical Package for the Social Sciences (SPSS; International Business Machines Corporation, version 29.0.2.0 [20]), and *p* < 0.05 was considered statistically significant. For multiple comparisons, the Bonferroni correction was applied to control for Type I error. In such cases, the adjusted p-value was calculated by multiplying the raw p-value by the number of tests performed [24].

## Results

### uPARAP expression in clinical STS and BS samples

**Fig. 1:**
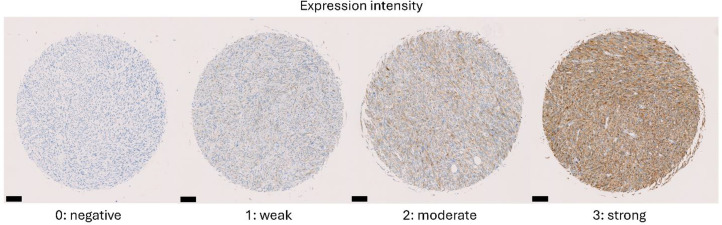
Examples of expression intensity scores from 0 to 3. Cores of tissues from the clinical leiomyosarcoma-specific tissue microarray.

**Fig. 2:**
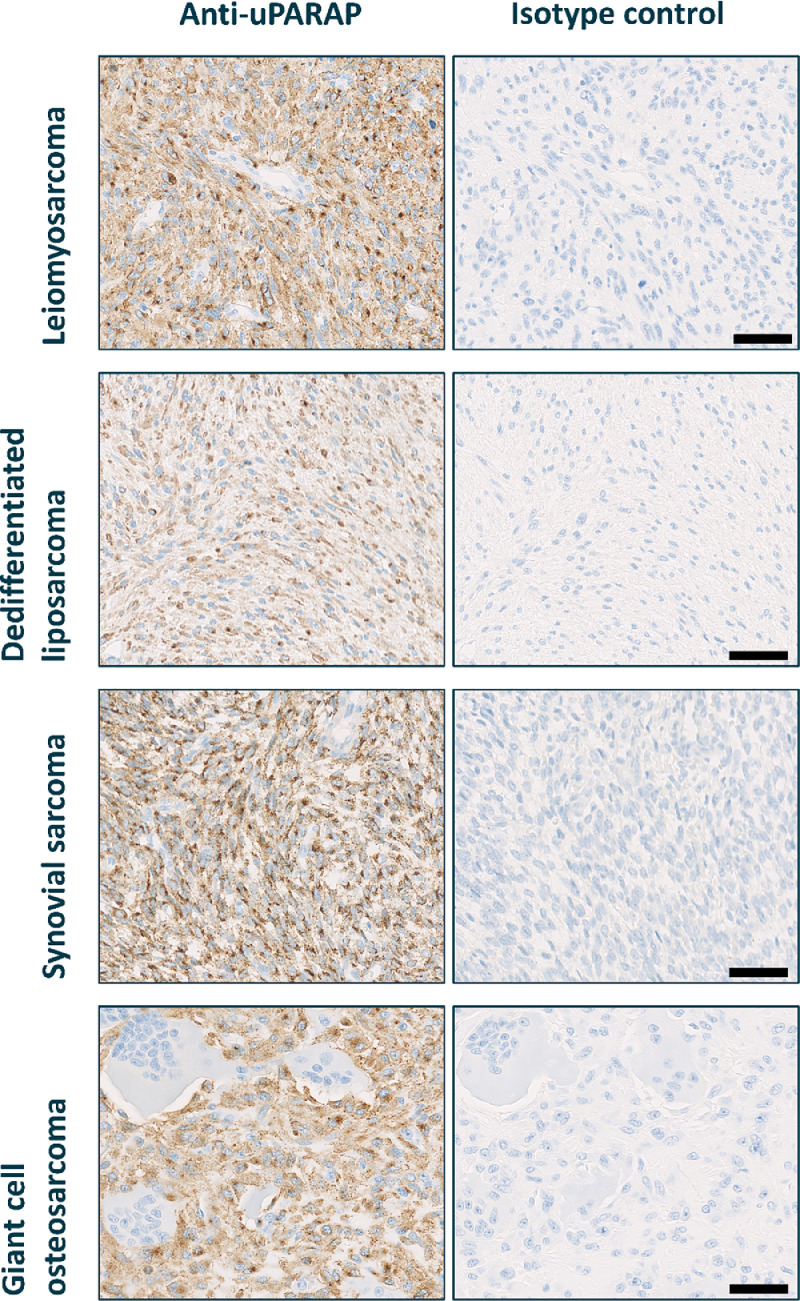
uPARAP expression in selected sarcoma cases compared to negative isotype control. uPARAP: urokinase plasminogen activator receptor-associated protein.

A total of 513 STS and BS samples were evaluated for uPARAP expression. An overview of expression levels across multiple sarcoma subtypes is summarized in Table 1. With few exceptions, all sarcoma subtypes showed high uPARAP expression in the majority of tumor samples (intensity score 2 or 3 and >50 % positive tumor cells). Notably, high expression was found in >80 % of cases in adult-type fibrosarcoma, SynSa, UPS, and MPNST. A few sarcoma subtypes demonstrated a more modest expression profile, with a higher proportion of cases showing low expression (intensity score 1 and/or <50 % positive tumor cells), such as WDLPS (43.8 % of cases), AS (54.5 %), and RMS (50 %). Furthermore, all cases of giant cell tumors, osteoblastoma-like osteosarcoma, and fibroblastic osteosarcoma showed high levels of uPARAP expression. However, the small sample sizes for osteoblastoma-like and fibroblastic osteosarcoma (*n* = 3 each) should be taken into account. Additionally, based on our analyzed cores, uPARAP expression was generally uniform across cores, with little evidence of frequent heterogeneous expression. Examples of cores with different intensity scores are shown in Fig. 1, and representative images of uPARAP IHC staining in highly expressing tumors compared to negative isotype controls are presented in Fig. 2.

**Table 1:** uPARAP expression in multiple sarcoma subtypes.

Sarcoma subtype	Cases (*n* = 513)	uPARAP Expression ( % of cases)^a^		
		High expression	Low expression	Negative
**Leiomyosarcoma**				
Primary tumor Metastatic tumor	58	46.6 %	37.9 %	15.5 %
	47	42.6 %	38.3 %	19.1 %
**Liposarcoma**				
Dedifferentiated	21	66.7 %	28.6 %	4.8 %
Pleomorphic	17	58.8 %	17.6 %	23.5 %
Myxoid round cell	26	46.2 %	38.5 %	15.4 %
Well-differentiated	16	25.0 %	43.8 %	31.3 %
**Fibrosarcoma**				
Myxofibrosarcoma	22	54.5 %	40.9 %	4.5 %
Adult-type fibrosarcoma	30	83.3 %	10.0 %	6.7 %
Dermatofibrosarcoma protuberans	54	92.6 %	7.4 %	0.0 %
**Synovial sarcoma**	107	81.3 %	15.0 %	3.7 %
**Undifferentiated pleomorphic sarcoma**	20	85.0 %	15.0 %	0.0 %
**Angiosarcoma**	44	25.0 %	54.5 %	20.5 %
**Rhabdomyosarcoma**	10	50.0 %	50.0 %	0.0 %
**Malignant peripheral nerve sheath tumor**	11	81.8 %	9.1 %	9.1 %
**Bone sarcoma**				
Osteosarcoma	11	72.7 %	27.3 %	0.0 %
Giant cell tumor	13	100.0 %	0.0 %	0.0 %
Osteoblastoma-like osteosarcoma	3	100.0 %	0.0 %	0.0 %
Fibroblastic osteosarcoma	3	100.0 %	0.0 %	0.0 %

a IHC staining scored by certified pathologists. High-expression: intensity score 2 or 3 and ≥ 50 % positive cells; low-expression: intensity score 1 and/or < 50 % positive cells; negative: < 5 % positive cells. IHC, immunohistochemistry; uPARAP, urokinase plasminogen activator receptor-associated protein.

Scalebar = 100 µm.

Scalebar = 50 µm.

### Correlation of uPARAP expression and selected clinical parameters

The correlation between uPARAP expression and selected clinical parameters was analyzed separately for 5 of the STS subtypes (AS, MFS, LMS, LPS, and SynSa) with available clinical information. LPS includes the 4 subtypes, DDLPS, WDLPS, MRCLS, and PLS. A total of 10 SynSa local relapse samples were excluded from the analysis, as all cases belonged to the same category (high expression group) due to the low number of samples, which could lead to misleading results. Samples lacking clinical information were excluded from the analysis when performing clinical correlations. MPNST, RMS, and UPS samples were excluded from the analysis due to small sample size.

As shown in Supplemental Tables 1–5, no significant association was found between uPARAP expression level and donor patient gender (female, male) across the different STS subtypes (AS, *p* = 0.187; MFS, *p* = 1.000; LMS, *p* = 0.272; LPS, *p* = 0.641; SynSa, *p* = 0.828). In AS, MFS, LPS, and SynSa samples, no statistically significant association was found between expression level and sample origin (primary tumor, local relapse, and metastatic lesions; AS, *p* = 0.100; MFS, *p* = 1.000; LPS, *p* = 1.000; SynSa, *p* = 0.908). However, in LMS samples, a borderline statistically significant association was observed (*p* = 0.050), suggesting a potential correlation between expression and sample origin. A higher percentage of cases with high expression were found in primary and local relapse samples, with 46 % (12/26) and 61 % (11/18) of cases, respectively. Adjusted p-values using the Bonferroni correction were considered.

### uPARAP expression and survival

**Fig. 3:**
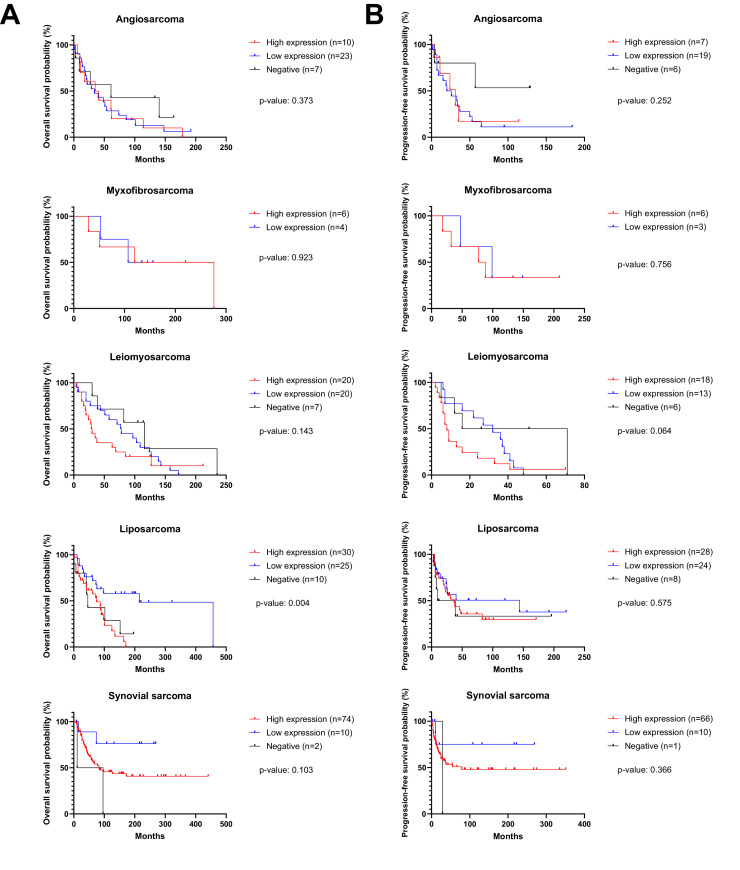
A) overall survival and B) progression-free survival according to uPARAP expression levels in different sarcoma subtypes. n: number of cases. Liposarcoma includes: differentiated, well-differentiated, myxoid round cell, and pleomorphic liposarcoma.

We investigated whether uPARAP expression has a prognostic role in the analyzed sarcoma subtypes. OS and PFS were calculated for each subtype and compared across different expression levels. As shown in Fig. 3, there appeared to be a direct correlation between high uPARAP expression and worse survival outcomes, suggesting a potential role for uPARAP as a prognostic marker in these STS subtypes. However, only in LPS (including DDLPS, WDLPS, MRCLS and PLS) was OS significantly associated with uPARAP expression (*p* = 0.004), with low expression correlating with better survival. The median OS was 215 months in the low expression group, compared to 75 and 46 months in the high and negative expression groups, respectively. In other STS subtypes, the correlations were not statistically significant.

### uPARAP expression in normal human tissues

**Fig. 4:**
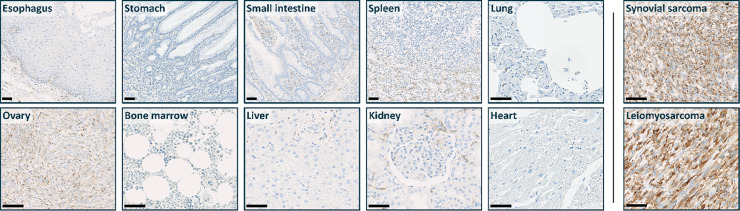
uPARAP expression in normal healthy tissues compared to tumor tissue from synovial sarcoma and leiomyosarcoma.

uPARAP expression was assessed in 32 types of normal human tissues (*n* = 3 for each tissue). In contrast to the high expression observed in STS and BS, much lower staining levels were found in normal tissues. Overall, uPARAP-positive staining was predominantly observed in interstitial and stromal cells across several tissues: weak staining in liver, neurohypophysis, salivary gland, skin and stomach; moderate staining in breast, cervix, colon, esophagus, kidney, larynx, lung, mesothelium, prostate and small intestine; strong staining in endometrium, ovary and testis (Fig. 4).

Scalebar = 50 µm in all images.

The epithelium was uniformly uPARAP-negative except for weak to moderate staining of basal epithelial cells observed in the esophagus and skin. In the kidney, staining of some glomerular tufts was observed. Occasional staining of cells adjacent to blood vessels was observed in the thymus, peripheral nerve, and skeletal muscle. Other observations included staining of zona reticularis and medullary regions of the adrenal gland, staining of sinusoids (red pulp) of the spleen, and staining in the geminal centers and interfollicular regions of tonsils.

## Discussion

Over the past decades, striking improvements have been made in the field of cancer research, and for some cancers (e.g., lung cancer, breast cancer, and melanoma), survival has improved significantly, mostly due to a better understanding of the cancer biology, earlier diagnosis, and novel, often targeted treatment strategies [[25], [26], [27], [28]]. In contrast, when examining the median OS for STS patients over the past 20 years (excluding gastrointestinal stromal tumors), survival for localized STS remained around 100 months (1999–2004: 105 months; 2005–2011: 97months) [25]. For metastatic STS, the median OS was 8–9 months (1999–2011: 8 months; 2012–2019: 9 months), with no significant improvement in survival for either localized or metastatic STS over the past decades, according to data from the Surveillance, Epidemiology, and End Results (SEER) database [25]. This highlights the urgent need for research in STS to improve our understanding of STS biology, to discover new actionable targets and to develop novel targeted therapies.

In this article, we report the expression of uPARAP in normal tissues, STS, and BS using clinical TMAs, with a total of 513 sarcoma samples analyzed. In addition, TMAs constructed at KU Leuven/UZ Leuven with available clinical data were analyzed to assess correlations between uPARAP expression and selected clinical parameters as well as clinical outcomes. Based on our findings, the vast majority of analyzed sarcoma subtypes showed high uPARAP expression, with particularly high numbers of cases in adult-type fibrosarcoma (83.3 % of cases), SynSa (81.3 %), UPS (85 %), MPNST (81.8 %), and various BS subtypes (72.7 %−100 %), while expression in normal tissues was generally weak and limited, highlighting the tumor-selective therapeutic potential of uPARAP. In the majority of analyzed STS subtypes, no significant association was found between uPARAP expression and either gender or sample origin, except in LMS, where a borderline significant correlation was observed between expression and sample origin (*p* = 0.050 after Bonferroni correction), suggesting that uPARAP expression could vary depending on the sample origin of LMS. In general, uPARAP expression did not show statistically significant correlation with survival (OS or PFS) in the analyzed sarcoma subtypes (AS, MFS, LMS, LPS, and SynSa). Even though OS was significantly associated with uPARAP expression in LPS (*p* = 0.004), this association might have been driven by the heterogeneity of LPS, as different subtypes were included.

Evans *et al*. have previously reported high levels of uPARAP expression in clinical samples from common STS subtypes, thereby emphasizing our findings [29]. Furthermore, according to previous studies, uPARAP has a high internalization rate, with receptors being internalized into cells within two minutes upon binding and efficiently recycled back to the cell membrane within 60 min [8,19,30]. This allows each receptor to mediate multiple rounds of uptake and prevents complete occupancy by collagen binding. Taken together, the high expression levels in sarcoma tumor cells, limited expression in normal tissues, and the receptor’s internalization and recycling further emphasize uPARAP as a highly promising therapeutic target for sarcoma treatment.

Antibody-drug conjugates (ADC), one of the fastest growing anti-cancer treatment modalities in the field of oncology recently, may be an ideal option to target uPARAP. ADC consists of a monoclonal antibody, a cytotoxic drug as payload, and a linker that connects the two. With a stable linker and an antibody that is target-specific and has high binding affinity (e.g., to uPARAP), the payload can be effectively delivered to the tumor site, while limiting normal tissue exposure. Therefore, compared to conventional systemic chemotherapy, ADC have the potential to improve efficacy while reducing systemic toxicity. In line with this, recent studies have demonstrated a promising cytotoxic effect of a uPARAP-targeting ADC in uPARAP-expressing cell lines and tumor models in mice, with no adverse side effects observed [8,29]. These results highlight uPARAP’s clinical potential and support the evaluation of uPARAP-targeted ADCs in sarcomas. Of note, a very first Phase I/II first-in-human study of a uPARAP-targeted ADC (ADCE-D01) in patients with metastatic and/or unresectable STS began in March 2025 and is currently recruiting patients in the United States and Europe (NCT06797999). The scientific rationale for this trial is in part based on the molecular epidemiology work reported here, the development of specific antibodies against the novel target and the exploration of such ADCs in mouse models in our laboratories [31].

In this study, all analyses were based on available TMA samples. The TMA technology allows for the screening of a large number of samples in a relatively short time and in a cost-efficient manner (e.g., reduced antibody usage). It is a very valuable tool in cancer research, such as for drug target discovery or genomic-based diagnostics, and is especially suitable when working with limited or precious tissue samples, for example, in rare diseases like sarcomas. On the other hand, this technology also has certain limitations. One commonly discussed concern is that the relatively small core samples included in the TMA may not be representative for the entire tumor, and thus, may not provide sufficient information about the sample as a whole, especially in cases of heterogeneous tumors. However, this limitation can be partially addressed by including multiple cores from different regions of the tumor and using larger core diameters (2–4 mm). If available, multiple samples from the same donor patient, collected from different sites or at different time points can also be included. Furthermore, it is important to note that the aim of TMA analysis differs from that of conventional histological analysis, as it is used as a populational-level research tool rather than for diagnostic purposes in individual clinical cases [32,33].

Another limitation of this study is that not all analyzed samples were included in the clinical correlations. Samples from the commercial TMA and those with missing clinical data were excluded for this part of the research. This highlights the limitations of using commercial TMAs in this type of study, as clinical information from donor patients and tissues may be incomplete or simply unavailable. Furthermore, the classification of tumor tissues in such commercial TMAs might not be updated according to the latest guidelines. This also underscores one of the major challenges in conducting research in orphan malignancies, particularly due to their rarity and heterogeneity, and emphasizes the importance of collaborative efforts in this field. In line with this, to support sarcoma research, the Laboratory of Experimental Oncology (KU Leuven, Belgium) has been establishing and expanding sarcoma research platforms, which include a clinical database linked to various well-annotated samples (e.g., blood, circulating tumor DNA, and TMA) as well as patient-derived xenograft models of sarcomas [22,34,35].

In conclusion, our study demonstrates high uPARAP expression across different sarcoma subtypes, the correlation of this expression with selected clinical parameters and outcomes, and the limited expression in normal human organ tissues. These findings highlighted the potential of uPARAP as a highly promising target for therapies such as ADCs and paved the way for further preclinical studies as well as the ongoing clinical evaluation of anti-uPARAP therapies in sarcomas.

## Funding sources

The study was partially funded by Adcendo ApS (Denmark) and the Laboratory of Experimental Oncology, KU Leuven (Belgium).

## Declaration of generative AI and AI-assisted technologies in the writing process

During the preparation of this work, the author(s) used OpenAI ChatGPT (GPT-5 version) to obtain grammar suggestions. After using this tool, the author(s) reviewed and edited the content as needed and take(s) full responsibility for the content of the publication.

## CRediT authorship contribution statement

**Chao-Chi Wang:** Writing – review & editing, Writing – original draft, Visualization, Validation, Software, Methodology, Investigation, Formal analysis, Data curation. **Pernille Barkholt:** Writing – review & editing, Writing – original draft, Visualization, Validation, Software, Methodology, Investigation, Formal analysis, Data curation. **Agnieszka Wozniak:** Writing – review & editing, Supervision, Resources, Project administration, Conceptualization. **Ulla Vanleeuw:** Writing – review & editing, Methodology. **Che-Jui Lee:** Writing – review & editing, Methodology. **Luna De Sutter:** Writing – review & editing, Methodology. **Lore De Cock:** Writing – review & editing, Methodology. **Kimberly Verbeeck:** Writing – review & editing, Methodology. **Lars Henning Engelholm:** Writing – review & editing, Conceptualization. **Carmel Lynch:** Writing – review & editing, Conceptualization. **Dominik Mumberg:** Writing – review & editing, Conceptualization. **Patrick Schöffski:** Writing – review & editing, Supervision, Resources, Project administration, Funding acquisition, Conceptualization.

## Declaration of competing interest

The authors declare the following financial interests/personal relationships which may be considered as potential competing interests:

P.S. has consulting or advisory role at Adcendo ApS, Boehringer Ingelheim, Boxer Capital, Cogent Biosciences, Deciphera, Ellipses Pharma, Exelixis, Guidepoint Global, IDRx, LLX solutions, Medpace, Merck Healthcare KGaA, NEC OncoImmunity AS, Oncode accelerator, SERVIER, Studiecentrum voor Kernenergie, Telix Pharmaceuticals, Thermosome, Transgene and UCB.

Received research funding from Adcendo ApS, Eisai, G1 Therapeutics, Merck, ONA Therapeutics, PharmaMar and Sartar Therapeutics.

P.B., L.H.E., C.L., and D.M. are employees of Adcendo ApS.

No conflict of interest declared by the other authors.

## Appendix Supplementary materials

Image, application 1
