# Supplementary material for: Molecular epidemiology of the expression of urokinase plasminogen activator receptor-associated protein (uPARAP) in mesenchymal malignancies
Source: Transl Oncol. 2026 Feb 23;66:102708. doi: 10.1016/j.tranon.2026.102708 (PMC13080477; doi:10.1016/j.tranon.2026.102708)
Supplement: Supplementary file 1 [file mmc1.docx]

**Supplemental Table 1**. Angiosarcoma; correlation of uPARAP expression with gender and sample origin.

| Patients and samples | Number of cases/ total number of cases with available information | uPARAP expression | | | p-value |
| --- | --- | --- | --- | --- | --- |
|  |  | High expression | Low expression | Negative |  |
| Donor patients (n= 40) | | | | | |
| Female | 26/40 (65%) | 4/26 (15%) | 16/26 (62%) | 6/26 (23%) | 0.187 |
| Male | 14/40 (35%) | 6/14 (43%) | 7/14 (50%) | 1/14 (7%) |  |
| Samples (n=37) | | | | | |
| Primary tumor | 22/37 (60%) | 5/22 (23%) | 13/22 (59%) | 4/22 (18%) | 0.100 ^*^ |
| Local relapse | 6/37 (16%) | 0/6 (0%) | 3/6 (50%) | 3/6 (50%) |  |
| Metastases | 9/37 (24%) | 4/9 (44.5%) | 4/9 (44.5%) | 1/9 (11%) |  |

^*^ Adjusted p-value using the Bonferroni correction (capped at 1.000 if it exceeds 1).

**Supplemental Table 2**. Myxofibrosarcoma; correlation of uPARAP expression with gender and sample origin.

| Patient and sample | Number of cases/ total number of cases with available information | uPARAP expression | | | p-value |
| --- | --- | --- | --- | --- | --- |
|  |  | High expression | Low expression | Negative |  |
| Donor patients (n=10) | | | | | |
| Female | 5/10 (50%) | 3/5 (60%) | 2/5 (40%) | 0/5 (0%) | 1.000 |
| Male | 5/10 (50%) | 3/5 (60%) | 2/5 (40%) | 0/5 (0%) |  |
| Samples (n=10) | | | | | |
| Primary tumor | 0/10 (0%) | 0/0 (0%) | 0/0 (0%) | 0/0 (0%) | 1.000 ^*^ |
| Local relapse | 3/10 (30%) | 2/3 (67%) | 1/3 (33%) | 0/3 (0%) |  |
| Metastasis | 7/10 (70%) | 4/7 (57%) | 3/7 (43%) | 0/7 (0%) |  |

^*^ Adjusted p-value using the Bonferroni correction (capped at 1.000 if it exceeds 1).

**Supplemental Table 3**. Leiomyosarcoma; correlation of uPARAP expression with gender and sample origin.

| Patient and sample | Number of cases/ total number of cases with available information | uPARAP expression | | | p-value |
| --- | --- | --- | --- | --- | --- |
|  |  | High expression | Low expression | Negative |  |
| Donor patients (n=47) | | | | | |
| Female | 30/47 (64%) | 9/30 (30%) | 17/30 (57%) | 4/30 (13%) | 0.272 |
| Male | 17/47 (36%) | 11/17 (64%) | 3/17 (18%) | 3/17 (18%) |  |
| Samples (n=97) | | | | | |
| Primary tumor | 26/97 (27%) | 12/26 (46%) | 11/26 (42%) | 3/26 (12%) | 0.050 ^*^ |
| Local relapse | 18/97 (19%) | 11/18 (61%) | 5/18 (28%) | 2/18 (11%) |  |
| Metastasis | 53/97 (54%) | 16/53 (30%) | 22/53 (42%) | 15/53(28%) |  |

^*^ Adjusted p-value using the Bonferroni correction (capped at 1.000 if it exceeds 1).

**Supplemental Table 4**. Liposarcoma; correlation of uPARAP expression with gender and sample origin.

| Patient and sample | Number of cases/ total number of cases with available information | uPARAP expression | | | p-value |
| --- | --- | --- | --- | --- | --- |
|  |  | High expression | Low expression | Negative |  |
| Donor patients (n=65) | | | | | |
| Female | 28/65 (43%) | 14/28 (50%) | 10/28 (36%) | 4/28 (14%) | 0.641 |
| Male | 37/65 (57%) | 17/37 (46%) | 15/37 (41%) | 5/37 (13%) |  |
| Samples (n=78) | | | | | |
| Primary tumor | 36/78 (46%) | 15/36 (42%) | 13/36 (36%) | 8/36 (22%) | 1.000 ^*^ |
| Local relapse | 25/78 (32%) | 12/25 (48%) | 11/25 (44%) | 2/25 (8%) |  |
| Metastasis | 17/78 (22%) | 8/17 (47%) | 7/17 (41%) | 2/17 (12%) |  |

^*^ Adjusted p-value using the Bonferroni correction (capped at 1.000 if it exceeds 1).

**Supplemental Table 5**. Synovial sarcoma; correlation of uPARAP expression with gender and sample origin.

| Patient and sample | Number of cases/ total number of cases with available information | uPARAP expression | | | p-value |
| --- | --- | --- | --- | --- | --- |
|  |  | High expression | Low expression | Negative |  |
| Donor patients (n=86) | | | | | |
| Female | 37/86 (43%) | 30/37 (81%) | 6/37 (16%) | 1/37 (3%) | 0.828 |
| Male | 49/86 (57%) | 44/49 (90%) | 4/49 (8%) | 1/49 (2%) |  |
| Samples (n=93) | | | | | |
| Primary tumor | 61/93 (66%) | 52/61 (85%) | 7/61 (12%) | 2/61 (3%) | 0.908 |
| Local relapse | Excluded from the analysis due to small sample size | | | |  |
| Metastasis | 32/93 (34%) | 27/32 (84%) | 4/32 (13%) | 1/32 (3%) |  |
